# Presence of pathogenic *Leptospira* spp. in the reproductive system and fetuses of wild boars (*Sus scrofa*) in Italy

**DOI:** 10.1371/journal.pntd.0008982

**Published:** 2020-12-28

**Authors:** Giovanni Cilia, Fabrizio Bertelloni, Ivana Piredda, Maria Nicoletta Ponti, Barbara Turchi, Carlo Cantile, Francesca Parisi, Paolo Pinzauti, Andrea Armani, Bruna Palmas, Malgorzata Noworol, Domenico Cerri, Filippo Fratini

**Affiliations:** 1 Department of Veterinary Science, University of Pisa, Pisa, Italy; 2 Istituto Zooprofilattico Sperimentale of Sardinia, Sassari, Italy; Universidade Federal de Pelotas, BRAZIL

## Abstract

Leptospirosis is a re-emerging and globally spread zoonosis caused by pathogenic genomospecies of *Leptospira*. Wild boar (*Sus scrofa*) are an important *Leptospira* host and are increasing in population all over Europe. The aim of this investigation was to evaluate *Leptospira* spp. infection in the reproductive systems of wild boar hunted in two Italian regions: Tuscany and Sardinia. From 231 animals, reproductive system tissue samples (testicles, epididymides, uteri) as well as placentas and fetuses were collected. Bacteriological examination and Real-Time PCR were performed to detect pathogenic *Leptospira* (*lipL32* gene). Leptospires were isolated from the testicles and epididymides of one adult and two subadult wild boar. Four isolates from the two subadult males were identified as *Leptospira interrogans* serogroup Australis by MLST, whereas *Leptospira kirschneri* serogroup Grippotyphosa was identified from the adult testicles and epididymis. Using Real-Time PCR, 70 samples were positive: 22 testicles (23.16%) and 22 epididymides (23.16%), 10 uteri (7.35%), 3 placentas (6.66%), and 13 fetuses (28.88%). Amplification of the *rrs2* gene identified *L*. *interrogans* and *L*. *kirschneri* species. The results from this investigation confirmed that wild boar represent a potential source of pathogenic *Leptospira* spp. Isolation of *Leptospira* serogroups Australis and Grippotyphosa from the male reproductive system and the positive Real-Time PCR results from both male and female samples could suggest venereal transmission, as already demonstrated in pigs. Furthermore, placentas and fetuses were positive for the *lipL32* target, and this finding may be related to a possible vertical transmission of pathogenic *Leptospira*.

## Author summary

Leptospirosis is an infectious zoonotic disease present worldwide and is caused by bacteria belonging to genus *Leptospira*. Several domestic and wild animals could serve as *reservoir* hosts of these bacteria and could transmit them to humans, directly or indirectly. Generally, in asymptomatic carriers the infection involves the urinary system, the kidneys in particular, and leptospires are eliminated *via* urine. In some animals, *Leptospira* could also colonize the reproductive system as consequence of a systemic infection or venereal transmission. In this work, the Authors describe, for the first time, the detection of *Leptospira* from male and female reproductive organs of hunted wild boar *via* culture-dependent and -independent methods. These findings could suggest a possible vertical transmission in wild boar, but also a possible venereal transmission cycle among wild swine, which could accidentally involve domestic pigs too. Manipulation of these organs by hunters during slaughtering could also expose them to *Leptospira* infection. Obtained data add information on *Leptospira* epidemiology and highlight the importance of wildlife monitoring programs.

## Introduction

Leptospirosis is a globally diffused, re-emerging zoonosis caused by pathogenic bacteria belonging to the genus *Leptospira* [1–3]. This includes 64 genomospecies; 38 of them are phylogenetically classified as pathogenic [4,5]. Pathogenic leptospires are responsible for infection in both humans and animals [1,6]. The primary site of *Leptospira* localization is the kidney, resulting in environmental spreading of the bacteria via urine and infection in a wide range of wild and domestics reservoir animals [6,7]. The genital tract could be secondarily affected after bacteremia and renal infection [8,9]. Recently, some studies carried out in cattle and pigs suggested that genital leptospirosis should be considered as a specific syndrome [7,10]. Genital leptospirosis seems to be related to low systemic antibody titers, and chronic uterine infection is often closely associated with a reproductive syndrome characterized by low fertility rates, abortion, and increased perinatal mortality. Sexual transmission has also been demonstrated [7,11–13]. Genital *Leptospira* infection has unique characteristics, which could be differentiated from renal disease [10]. In the Suidae family, especially in pigs, genital leptospirosis has been observed in sows, sometimes associated with abortion, and in boars [12,14,15]. Moreover, *Leptospira* infection of the reproductive tract has also been demonstrated in feral swine with the isolation of *Leptospira borgpetersenii* serogroup Ballum in wild boar aborted fetuses [16].

In Europe, wild boar (*Sus scrofa*) represent one of the most widespread large mammals [17,18]. This species is very adaptable and is able to survive in different habitats, including suburban and urban areas [17,19,20]. In Italy, the wild boar population is widespread, from the Alps to the southern part of the Italian peninsula, including the islands [18,21].

The reproductive cycle of wild boar is characterized by early puberty (from 5 to 10 months of age), high fertility, and short gestation period (from 115 to a maximum of 122 days) [22–25]. The estrous cycle has a duration of about 22 days, with receptivity for 1–3 days and usually one mating season per year. During the reproductive season, which is in Autumn when the rainfall increases, as well as the leptospirosis infection risk, males generally mate with more than one female [24–26]. Births usually occur in late winter to early spring, with a peak in February or March [26–28]. Furthermore, a bimodal distribution has been observed in some years, as a consequence of genetic breeding with domestic pigs [25,27]. Wild boar reproductive parameters could be affected by habitat, climatic conditions, photoperiods, hunting pressure, and availability of food resources [25,28,29].

The aim of this investigation was to evaluate the possible genital *Leptospira* infection in male and female wild boar. Moreover, the possibility of vertical transmission to fetuses was evaluated.

## Materials and methods

### Sample collection

During the authorized hunting season 2018/2019 (October–January) according to the regional Italian hunting Law (Regolamento di attuazione della legge regionale 12 gennaio 1994, n. 3 D.P.G.R. 48/R/2017 –Regione Toscana and Legge Regionale 29 luglio 1998, n. 23 –Regione Sardegna), wild boar tissue samples from the reproductive system were collected in two Italian regions: Tuscany and Sardinia. Since sampling was conducted during the hunting season, the sample size could not be predicted beforehand. Testicles, epididymides, and uteri were collected as well as placentas and fetuses from pregnant wild boar. The fetuses from each pregnant wild boar were pooled and considered as a single sample. Organs were collected by veterinarians during slaughtering, performed by hunters, minimizing all possible contamination. The age of each hunted wild boar was determined after assessing the degree of tooth eruption and wear and tear of teeth of the lower jaw [30]. Three age classes were considered: young (under 12 months), subadult (between 12 and 24 months), and adult (over 24 months). The sex of hunted animals was also recorded. No animals were specifically sacrificed for this study purpose.

### *Leptospira* spp. isolation and MLST genotyping

Organs were processed under a biological safety hood using sterile forceps, scissors, and scalpels. External capsules of testicles and epididymides were carefully removed, and internal tissues were collected using a different set of sterile instruments for each organ. Concerning female organs, the uterus was carved, and internal mucosa, placenta, and (when present) fetuses were collected using a different set of sterile instruments for each sample.

For each tissue sample, three to five distinct portions of approximately 1 cm^3^ were collected and homogenized with 5 ml of sterile water in a Stomacher 400 Circulator (Seward LTD, West Sussex, United Kingdom). One milliliter of the homogenate was serially diluted in three tubes containing 5 ml of Ellinghausen-McCullough-Johnson-Harris (EMJH) media (Difco, Detroit, MI, USA) to perform the cultures. Tubes were then incubated at 30°C ± 1°C for 120 days and observed every 10 days under dark-field microscopy to evaluate possible bacterial growth. In cases of overgrowth of other microorganisms, 2 ml from the contaminated tubes were filtered using 0.20 μm pore size filters and then sub-cultured in new EMJH media at the previously described conditions. Tubes were discarded if this procedure was not able to prevent further contamination.

The obtained *Leptospira* isolates were genotyped using a Multilocus Sequence Typing (MLST) scheme based on housekeeping genes [31].

### *Leptospira* spp. detection by multiplex Real-Time PCR and genomospecies identification

DNA was extracted from each tissue sample using the Quick-DNA Plus Kit (Zymo Research, Irvine, CA, USA) following the manufacturer’s instructions. All samples were tested by multiplex Real-Time PCR, identifying the *Leptospira* genus-specific target located on *16S rRNA* gene and the specific target for pathogenic species (*lipL32* gene) [32,33]. The Real-Time PCR assay was performed on a Rotorgene Corbett 6000 (Corbett Research, Sidney, Australia) with the following thermal conditions: a holding stage of 95°C for 5 min, 45 cycles of 95°C for 15 sec, and 60°C for 30 sec. Samples were considered positive with a Ct < 40.

Finally, from PCR-positive tissue samples, *Leptospira* species were identified using a primer for the *rrs2* gene [34]. Amplification of each target gene was carried out using a HotStarTaq Master Mix Kit (Qiagen, Hilden, Germany). Amplicons were further sequenced (BMR Genomics, Padova, Italy) using the same amplification primer sets and analyzed using BioEdit Software [35]. Phylogenetic analysis based on the *rrs2* gene was performed using the Maximum Likelihood method based on the Tamura-Nei model using MEGA 10 software [36].

### Statistical analysis

Data were analyzed with Chi-square (*X*^2^) and Fisher (F) tests. Statistical tests were used to evaluate the *Leptospira* infection ratio regarding sex (male or female), age (young, subadult, or adult), pregnancy (pregnant or not), and provenience (Tuscany or Sardinia) of the wild boar. In addition, stratified analyses were performed considering all the possible parameter combinations (i.e., only males stratified by age). The statistical significance threshold was set at a *p* value ≤ 0.05 [37].

## Results

### Sample collection

During the hunting season, 231 wild boar specimens were sampled: 200 from Tuscany and 31 from Sardinia. Ninety-five were males and 136 females (45 were pregnant), subdivided in 110 adults, 40 subadults, and 81 young animals. All hunted animals did not present macroscopic lesions related to leptospirosis during the *post-mortem* examination.

### *Leptospira* spp. isolation, identification, and MLST genotyping

After about 70 days of incubation, six *Leptospira* isolates were obtained from three testicles and the respective epididymides of an adult male and two subadults (Table 1). Isolates from adult male tissues were identified as *Leptospira kirschneri* serogroup Grippotyphosa and assigned to Sequence Type (ST) 78, while the remaining four isolates from two subadult males were identified as *Leptospira interrogans* serogroup Australis and assigned to ST 24 by MLST analysis.

**Table 1:** Distribution of positive tissue samples for pathogenic *Leptospira* in relation to sex and age class.

Sex	Age Class	No. of animals	Bacteriological positive samples	PCR-positive tissues			
				Testicles and Epididymides	Uterus	Placentas	Fetuses
Male (n = 95)	Adult	37	1	7	-	-	-
	Subadult	22	2	6	-	-	-
	Young	36	0	9	-	-	-
Female	Adult [Pregnant]	73[45]	0[0]	-	9[5]	[3]	[13]
(n = 136)	Subadult	18	0	-	1		
	Young	45	0	-	1		

### *Leptospira* spp. detection by multiplex real-time PCR and genomospecies identification

*Leptospira* DNA was detected in male and female tissues samples. Data reported in Table 1 highlight that 22 out of 95 (23.16%) testicles and the respective epididymides scored positive by Real-Time PCR. Eleven out of 136 (8.08%) uterine samples (five of them from pregnant females, 3.68%), 3 placentas (6.66%), and 13 fetuses (28.88%) out of 45 were positive by Real-Time PCR (Table 1).

**Fig 1:**
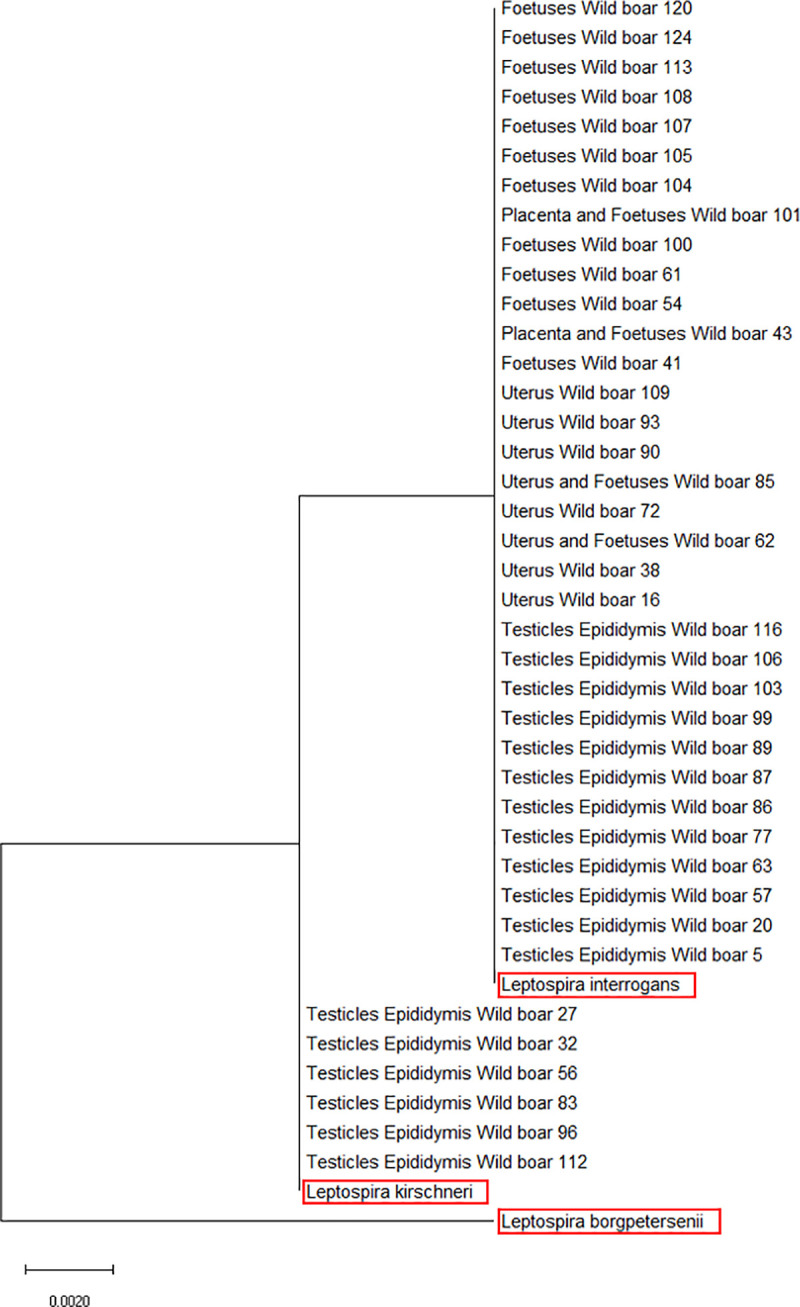
Molecular phylogenetic analysis for PCR-positive tissue samples based on the *rrs2 gene* of *Leptospira interrogans*, *Leptospira borgpetersenii*, and *Leptospira kirschneri* (in the red box with the GenBank Accession Number) by the Maximum Likelihood method based on the Tamura-Nei model. The branch lengths of the tree measure the number of substitutions per site. The analysis involved 31 nucleotide sequences. There was a total of 452 positions in the final dataset.

Regarding the characterization of PCR-positive samples, amplification of the *rrs2* gene highlighted that pathogenic *Leptospira* belonged to *L*. *interrogans* and *L*. *kirschneri*. Phylogenetic analysis identified a close relationship between the respective *Leptospira* species (Fig 1).

### Statistical analysis

No statistical difference (p>0.05) was recorded on the prevalence of *Leptospira* infection considering sex, age, pregnancy, and provenience of all the wild boar, in total, and for each provenience region. Moreover, no statistical differences were detected comparing *Leptospira* infection rates in Tuscany and Sardinia regions, considering sex, age, and pregnancy.

## Discussion

In this study, L*eptospira* infection in the reproductive tract of male and female wild boar was evaluated by culture-dependent and -independent methods. DNA of pathogenic *Leptospira* was detected in samples from both males and females, as well as in all age classes, confirming endemic infection among wild boar in the investigated areas. The results of this investigation suggest that both genders and all age classes have the same probability to be infected with *Leptospira*. However, no suggestive macroscopic lesions were observed during *post-mortem* examination. During the last decades, the wild boar population has rapidly increased in Italy. This species is adapted to live in contact with humans (i.e., hunters, farmers, etc.) and domestic animals, hence promoting the possibility of interspecies leptospirosis transmission [17,38].

To the best of the Authors’ knowledge, no studies have focused on involvement of the reproductive system in *Leptospira* infection in wild boar. Based on the obtained data, all positive males presented infected testicles and epididymides, suggesting dual localization. This was confirmed by cultural examinations. *Leptospira* infection in the reproductive system has already been demonstrated in pigs [7,15]. *Leptospira interrogans* serovar Bratislava and serovar Muenchen were indeed isolated from swine testicles and urogenital tracts [15]. This investigation suggested that male wild boar could be infected with swine-adapted pathogenic *Leptospira interrogans* serovar Bratislava.

The presence of *Leptospira* in the epididymis might be temporary and a consequence of testicular infection; however, there is no available detailed information clarifying this aspect in other domestic or wild animals.

Generally, as reported for bulls and boars, infections in males are often subclinical [7,10,39]. However, during the acute phase of genital infection, male wild boar could present mild and temporary orchitis. Testicular and/or epididymis inflammation due to leptospirosis could cause damage or alter sperm; however the specific damage caused by *Leptospira* infection to spermatozoa or its influence on sperm viability remains unknown. On the other hand, for other species, the possibility of venereal transmission from males to females is well documented. Pathogenic *Leptospira* in semen are able to travel up the reproductive tract and reach the uterus and oviduct, as reported in cattle [10,40]. However, although the role of semen in the transmission of bovine leptospirosis is well established [41,42], its role in transmission between wild and domestic swine has not been investigated and can only be hypothesized.

A commercial medium, without modifications, was used for *Leptospira* detection. After more than 2 months of incubation, serovar Bratislava was successfully isolated. Indeed, it is known that the isolation of serovar Bratislava is challenging, sometimes requiring a longer incubation period and use of a specific medium (EMJH with the addition of Tween 80, Tween 40, and lactalbumin hydrolysate) [12,14,43]. For this reason, the differences between the prevalence obtained by cultural methods and PCR could also be due in part to the medium employed.

In Tuscany, a high seroprevalence of serogroup Grippotyphosa has been recorded among cattle, sheep, and goats [44]. In Sardinia, wild boar are often exposed to *Leptospira* Bratislava and Pomona, thus potentially acting as a maintenance host for these serovars [45]. Consequently, the observed infection with *Leptospira kirschneri* serovar Grippotyphosa in an adult male wild boar could be due to free-ranging wild boar sharing their habitat with domestic ruminants.

The wild boar uterus seems to be receptive to *Leptospira* infection, not only in adults but also in young animals. Some specific *Leptospira* strains could be adapted to the uterine environment of wild boar, as already hypothesized for cattle. Indeed, it was recently speculated that infection by strains of the Sejroe serogroup in cattle, causing subclinical disease, is associated with strains presenting a certain level of adaptation to the reproductive tract [10]; in this case, a chronic, silent infection occurs and is characterized by agalactia in lactating animals and reproductive failures rather than abortion. It was also suggested that this genital adaptation could promote venereal transmission from females to males [7,10]. Considering that in this investigation no alteration was recorded, the presence of *Leptospira* in fetuses could suggest a high adaptation level of *Leptospira* strains and wild boar resistance to infection, which could promote vertical transmission without obvious signs in fetuses. On the other hand, for domestic swine, it was observed that some serovars could influence pregnancy, causing abortions, stillbirths, or birth of weak piglets with reduced viability [7,14]. As suggest for bovines, these events could be related to non-adapted serovars [7,10]. Unfortunately, little information is available clarifying this aspect. The only report concerning this issue reported the isolation of *Leptospira borgpetersenii* serogroup Ballum from wild boar aborted fetuses. This serogroup was not detected in this present survey by isolation and molecular investigations [16].

While the body of knowledge concerning wild boar is limited, several studies on pathogenic *Leptospira* from sow reproductive systems are available [7,14,43]. *Leptospira* belonging to serogroup Australis was isolated from the uterus of sows [14,43], and infections with serovar Bratislava and Muenchen could be associated with abortions in sows [12,14,43].

In this study, no correlation among positive uterus, placenta, and fetus specimens was detected in pregnant females. This is in accordance with a previous study performed on pigs with a history of abortion, where no correlation between positive fetuses and placentas was found [12]. In animals, abortion could be a consequence of uterus inflammation or the fetuses themselves. In this study, the highest prevalence of *Leptospira* infection was detected in fetuses, followed by the uterus and placenta. As previously suggested for swine [12], *Leptospira* infection in female wild boar could persist and could be fatal to fetuses during pregnancy. Considering the obtained results, it might be hypothesized that pathogenic *Leptospira* could infect the kidneys, liver, or other fetal tissues. Unfortunately, based on the present data, it is not possible to establish whether fetuses would be delivered immunized, infected, or would be aborted.

According to phylogenetic analysis, pathogenic *Leptospira* DNA detected in wild boar reproductive systems belonged to *L*. *kirschneri* and *L*. *interrogans*. *Leptospira kirschneri* infection seems to be prevalent in testicles and epididymides, while *Leptospira interrogans* is present in female genital organs. Based on the serovars more often detected in Italy by cultural examination or serology [46–52], and the rare seropositivity for other serovars [53–55], it is likely that the detected *L*. *kirschneri* belonged to the serogroup Grippotyphosa. In this study, *L*. *kirschneri* infection was only observed in testicles and epididymides, and this could hypothesize this genomospecies as more often associated with the male reproductive system. Moreover, considering the negativity in females, it could be speculated that venereal transmission of *L*. *kirschneri* is unlikely within wild boar. In addition, the ability of this *Leptospira* species to survive and colonize the uterus compared to other species should be assessed.

As concerns the presence of *L*. *interrogans*, it would be very difficult to infer the serogroup since this genomospecies includes several groups, such as Icterohaemorrhagiae, Canicola, Pomona, Australis, and Sejroe. However, in relation to isolates obtained in this investigation and previously published serological results [44,46,51,56], it is likely that serogroup Australis would be prevalent, at least in samples from males. Currently, serovar Bratislava and the closely related serovar Muenchen, belonging to serogroup Australis, are the main emerging swine-maintained pathogenic *Leptospira* serovars [7]. The epidemiology of these serovars is poorly clarified; however, their strains are grouped into specific pig-adapted strains, strains maintained by pigs, dogs, horses, and hedgehogs, and strains found only in wildlife [7,57]. In pig farms, venereal transmission seems to play an important role in the spread of serovars belonging to the Australis serogroup, especially for Bratislava. In this context, frequent introduction of new male subjects to the farms could represent an important risk factor [9,12,15]. Therefore, genital infection with serovar Bratislava in wild boar could suggest the possibility of venereal transmission among the wild swine population and, on the other hand, increase the risk of transmission to domestic pigs via possible mating with wild boar, especially in areas where semi-extensive breading systems are adopted such as Tuscany or Sardinia.

This is the first investigation focusing on the isolation of pathogenic *Leptospira* from the reproductive system of healthy wild boar. The impact of *Leptospira* infection on the reproductive system and performance of wild boar is of great interest since leptospirosis is a neglected and underestimated disease and has an economic impact on the breeding system. The results obtained contribute to understanding the role of wild boar as a pathogenic *Leptospira reservoir*, and potentially a spreader as well. *Leptospira* could also cause silent and chronic infection, increasing the possibility of leptospirosis spreading among wild boar population. Unfortunately, in a natural system, the control of genital leptospirosis, and its effects on wild boar reproductive performance, is a challenging task. Under the same conditions experimental infection could be very difficult to perform as well. Conversely, the occurrence of infection is easily assessed in specimens from hunted animals, which is only a snapshot of the entire wild boar population for an investigated area. Data from such investigation could be compared to those on wild boar population dynamics; in this case, only long-term surveillance, also excluding other causes of reproductive syndromes in animals, could probably give exhaustive information.

*Leptospira* infection in the reproductive system of wild boar could be involved in the epidemiology of human leptospirosis. The hunters’ risk of exposure could be increased during slaughtering, since tissues from the reproductive system are necessarily handled during slaughtering, and the use of personal protective equipment is not commonly adopted among hunters.

In conclusion, the results of this investigation indicate that the genital tract of wild boar could be a target for different *Leptospira* species. Genital leptospirosis could be associated with reproductive problems, as reported in pigs. Wild boar are more resistant than selected breeding lineages, and they are not subjected to the stresses of intensive farming; for these reasons, the impact of genital leptospirosis on reproductive performance could be lower than in domestic swine, as suggested by the high birth rates reported recently in Italy as well in Tuscany and Sardinia [18,21]. Moreover, the pathogenic *Leptospira* localization in the reproductive systems could represents a public health risk, especially for hunters, that usually handled these organs during the slaughtering practices without the use of personal protective equipment, favoring the possible contagion.

The possible vertical transmission of *Leptospira*, suggested by the obtained results, opens new questions on the epidemiology of this disease in wild boar. Further investigation should be performed in order to understand the prevalence of *Leptospira* in the reproductive system and fetuses, the possible implication of genital infection on reproductive performance, and the effects of vertical transmission and consequent congenital infection, not only in wild boar but also in other wild animal species.
